# Preputial Calculus: Unveiling a Rare Encounter and Treatment Journey

**DOI:** 10.7759/cureus.58968

**Published:** 2024-04-25

**Authors:** Dheeraj Surya, Pankaj Gharde, Kavyanjali Reddy

**Affiliations:** 1 General Surgery, Jawaharlal Nehru Medical College, Datta Meghe Institute of Higher Education and Research, Wardha, IND

**Keywords:** preputial calculus, urological malformations, phimosis, obstructive uropathy, hematuria, preputial stones

## Abstract

Preputial calculus is an infrequent manifestation of urolithiasis, primarily observed in ageing individuals with an uncircumcised penis and not maintaining proper hygiene, which can further be complicated by co-morbidities such as phimosis. On the contrary, phimosis and other neurological/urological malformations have also been reported in children to cause preputial calculus. Overall clinical presentations include a palpable mass within the prepuce, dysuria, hematuria, obstructive uropathy, diminished urine flow, and malodorous discharge. This is a case of a 65-year-old male presented with a complaint of obstructed urinary flow. The patient was diagnosed with obstructive uropathy due to the presence of preputial stone/s. This case illustrates both singular and multiple stones in the affected patient. The patient was managed by surgical intervention by circumcision followed by calculus removal. As per the available published literature, this case can be noted as the first report of the largest preputial stone in an elderly in any rural setup of central India.

## Introduction

Preputial Calculus, also known as smegma stones or preputial calculi can be defined as the accumulation of the calcified debris in the preputial sac. This is a rare clinical condition, which was first reported by Robert Clarke in 1794. It is a form of urolithiasis, which has been found to be under-reported majorly due to its rarity and occurrence in underdeveloped countries [1]. While relatively rare, preputial calculi can lead to slight discomfort, penile swelling, and even complications if left untreated. Calculi are easily detected through clinical examination due to their free movement within the preputial sac [2]. In addition to clinical assessment, plain radiography serves as a valuable diagnostic tool. The diagnosis of preputial calculi can be confirmed by the radio-opaque lesions. The rest of the urinary tract can be evaluated for the presence of any other calculi for further treatment and management [3]. Despite its infrequency, awareness of this condition is crucial for timely intervention. The absence of retraction ability of the foreskin is defined as Phimosis, which can predispose individuals to the accumulation of debris and subsequent calcification within the preputial space. The co-existence of preputial calculus and phimosis further complicates clinical management, often necessitating tailored approaches to diagnosis and treatment. This case underscores the importance of addressing preputial calculus comprehensively to prevent long-term sequelae and optimize patient outcomes.

## Case presentation

**Figure 1:**
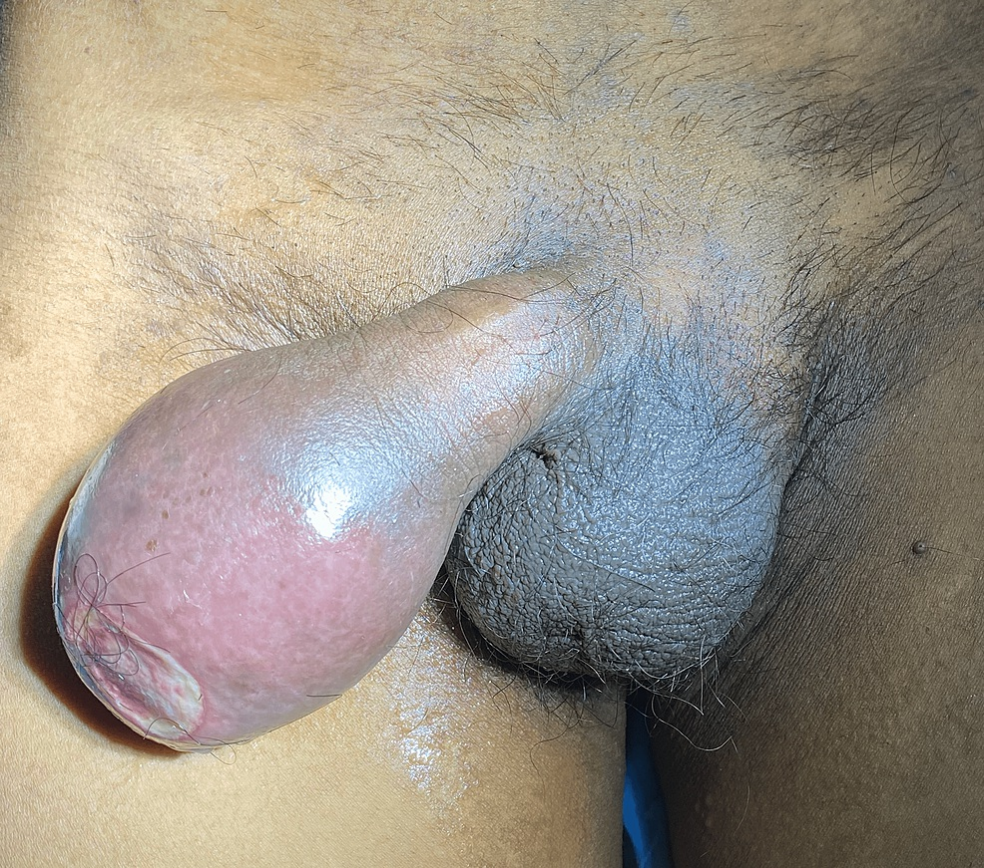
Clinical presentation of the distorted penis with enlarged prepuce with phimosis

A 65-year-old male visited the casualty department of a rural medical college in Maharashtra, India, with major complaints of pain in hypogastrium with urinary retention for one day. The patient did not have any significant medical or surgical history. On admission, the patient was vitally stable. The abdomen examination showed a soft and tender abdomen. The pelvic region showed palpable bladder without any rigidity and guarding and normal bowel habits until the previous day of admission. Lab investigations were found to be within normal limits. Clinical examination of the genitalia showed a distorted and enlarged prepuce afflicted with phimosis (Figure 1).

**Figure 2:**
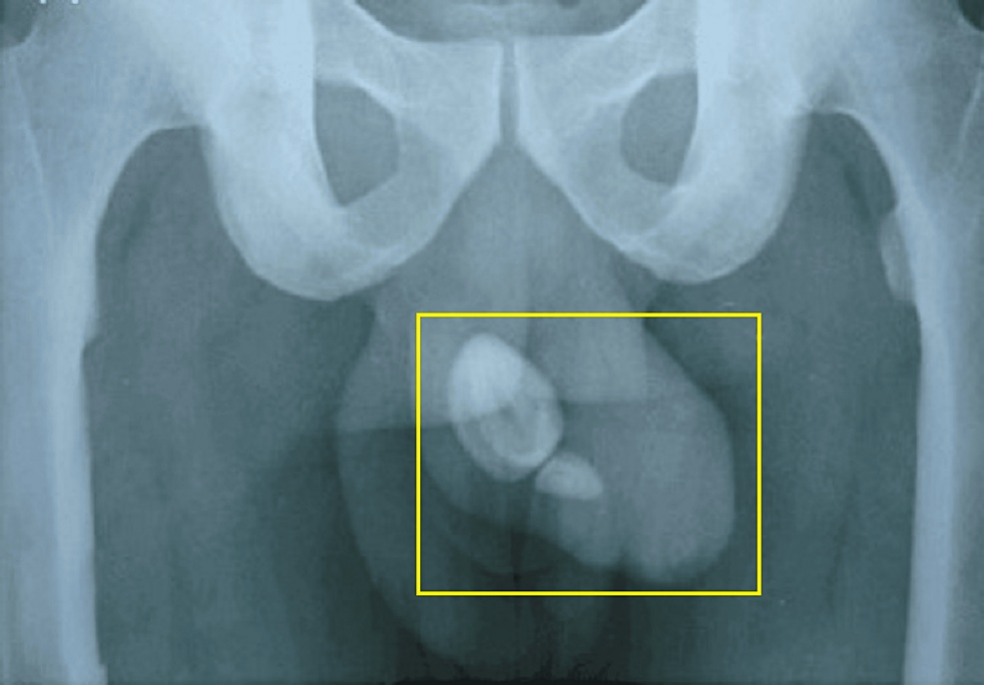
Radiology image of the patient showing a radiopaque lesion in his penile region The highlighted area shows radiopaque lesions indicative of preputial calculi.

A sizable, stony-hard foreign object, measuring 5 × 5 cm, was incidentally discovered beneath the prepuce. The testes appeared normal and were palpable bilaterally. The patient was unable to retract prepuce from the past 15 years and was unaware of any other clinical symptoms. The patient had no history of genital trauma, hematuria, or pyuria and passed pale yellow-coloured urine. The patient consulted a general physician three years ago for a poor stream of urine and was advised conservative management with oral medications but the documents were not available for the same. Sexual life continued till the age of 42 years with a single sexual partner. Subsequently, the patient underwent an X-ray imaging which revealed a radiopaque lesion with well-defined margins in his penile region, which was suggestive of a large stone (Figure 2).

**Figure 3:**
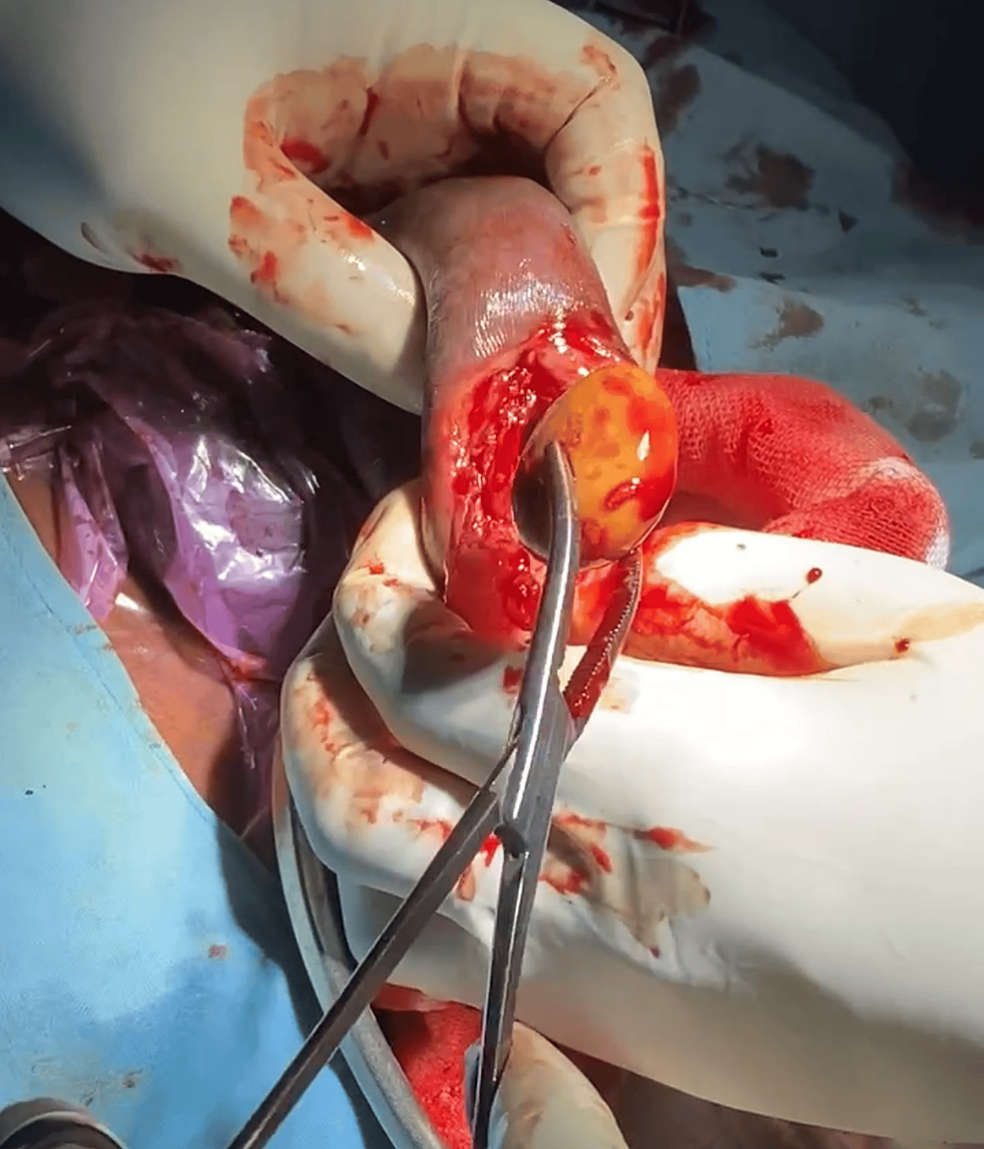
Intraoperative image of the penile skin incision revealing multiple calculi

**Figure 4:**
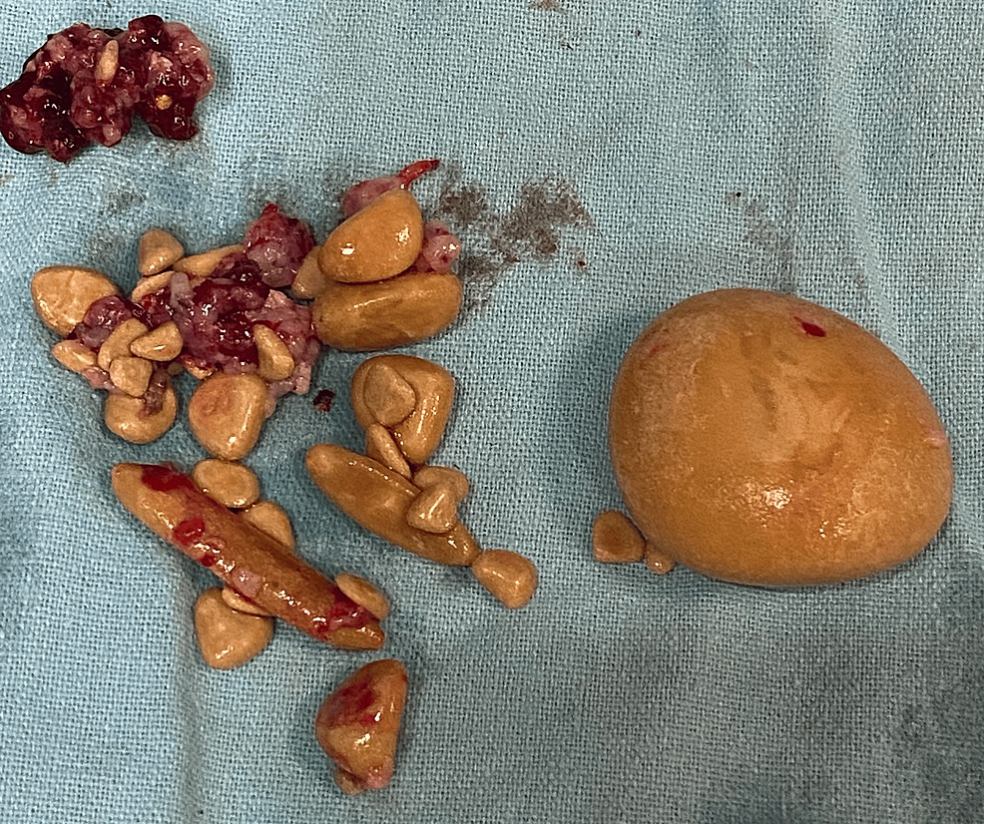
Multiple stones of different sizes extracted from the patient

**Figure 5:**
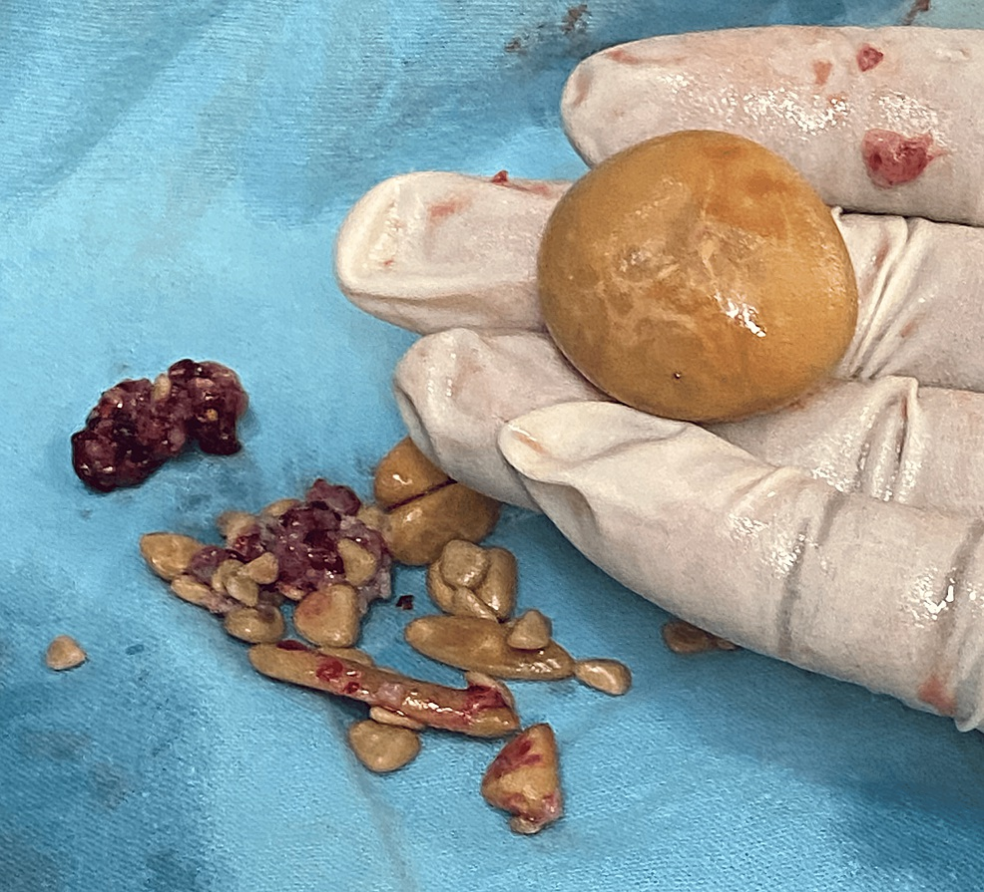
Largest stone extracted from the patient The dimensions of the largest stone were 3.0 x 2.0 cm.

A detailed ultrasonography (USG) of the abdomen and pelvis was done to rule out renal calculi and showed no signs of renal or ureteric calculus. The patient was planned for surgical management by circumcision along with the removal of the preputial stone. The circumcision procedure utilized a dorsal slit technique, through which the massive preputial stone measuring 5.0 x 5.0 centimeters was safely extracted without any complications. Along with the extraction of 15-20 multiple stones of different sizes, the largest of which measured 3.0 x 2.0 centimeters, the longest 3.0 x 0.5 centimeters, and multiple small tiny calculi of different sizes ranging between 3.0-5.0 millimeters were removed (Figures 3-5).

Post-surgery cystoscopic examination revealed no urethral stricture and a trabeculated bladder. Initially, the serum creatinine levels were noted to be raised, but they were found to be within normal limits post hydration. The calculus was not sent for chemical analysis due to financial constraints. The patient had a good recovery and no post-operative complications were noted. The patient was discharged post-operation on day 5 with Foleys in situ.

## Discussion

Large preputial stones are not so common, with common clinical presentations found to be obstructive uropathy and acute urinary retention. Such cases coexisting with phimosis underscores several important clinical considerations. Firstly, the presence of phimosis predisposes individuals to the accumulation of debris within the preputial sac, creating an environment conducive to the formation of calculi [1,3]. Winsbury-White has reported three underlying mechanisms for the formation of preputial calculi as inspissated smegma, a combination of smegma and urinary salts, and lastly the concentration of the urinary slats alone. Another research by Wilford has presented the pathogenesis characterization, which also lists inspissated smegma with lime salts along with other underlying reasons, such as struvite composition secondary to infection and the stones formed in the proximal urinary tract trapped during migration [1]. The underlying pathology, in this case, can be precipitated conjointly to the accumulation of precipitated urinary salts and inspissated smegma in the uncircumcised penis. Inflammation can also be attributed to the irritant potential of the smegma itself, which can secondly, lead to diagnostic challenges [2,4]. Differential diagnosis may include infections, inflammatory conditions, and neoplastic processes. Hence, a thorough history, physical examination, and imaging studies are essential for accurate diagnosis and management. Thirdly, its management requires a tailored approach involving the management of the predisposing cause with the extraction of the calculi. Conservative measures such as topical hygiene and gentle foreskin manipulation may be attempted initially to alleviate symptoms and facilitate calculus dissolution [4]. However, surgical intervention may ultimately be necessary, especially in cases of recurrent symptoms or complications such as urinary obstruction or secondary infections, which was similarly carried out in this case which was managed by the circumcision and calculi extraction by dorsal slit method [5]. Though preputial calculi are not life-threatening, they are recommended to be timely addressed, failing which serious complications such as obstructive uropathy, hydronephrosis, preputial fistula, and even renal failure might be noted [2,6]. Furthermore, the case highlights the significance of patient awareness and counselling in relation to penile hygiene practices to prevent the recurrence of preputial calculus and associated complications.

## Conclusions

Understanding the significance of personal hygiene is crucial, particularly for individuals who are uncircumcised or have neurological impairments. It's important to promote circumcision and stress the importance of maintaining good hygiene to the public. It's also worth noting the lack of education on healthy genital hygiene practices in our country since adolescence, highlighting the need for more comprehensive education on this matter. Early detection and timely intervention are keys to preventing the onset of complications mentioned earlier. Hence, the management requires a multidisciplinary approach, involving urologists, primary care physicians, and patient education. Timely diagnosis and treatment are vital for a good prognosis and prevention of long-term sequelae.
